# MRSA endocarditis of bovine Contegra valved conduit: a case report

**DOI:** 10.1186/1757-1626-2-57

**Published:** 2009-01-15

**Authors:** Gani Bajraktari, Rozafa Olloni, Irfan Daullxhiu, Fadil Ademaj, Zana Vela, Mubekir Pajaziti

**Affiliations:** 1 Service of Cardiology, Internal Medicine Clinic, University Clinical Centre of Kosovo, Prishtina, Kosovo; 2 General Hospital "Isa Grezda", Gjakova, Kosovo

## Abstract

**Background:**

Different techniques are used for the right ventricular outflow tract reconstruction, including homo- or porcine xenografts, which have several limitations. Contegra, a bovine jugular vein graft, is an interesting alternative to overcome these limitations. It consists of a bovine jugular vein with a naturally integrated valve in it. Isolated pulmonary valve endocarditis is extremely rare.

**Case presentation:**

We report the case of a 20 years old male patient with acute endocarditis of bovine Contegra valved conduit, four years after right ventricular outflow tract reconstruction and atrial septal defect correction, associated with acute glomerulonephritis, renal failure and severe anemia, secondary to methicillin-resistant Staphylococcus aureus infection (MRSA).

**Conclusion:**

We present a complex patient with acute endocarditis of bovine Contegra valved conduit. We believe that the presentation of this case should encourage the researchers for the discussing of the implantation of this conduit and the prevention of endocarditis in these patients.

## Background

A variety of prosthetic conduits and homografts for the right ventricular outflow tract reconstruction have been developed in recent decades. Homografts, which have been presented as the most reliable option, have shown early degeneration and calcification, particularly in very young patients [1], and those failed to be the best choice in the long term follow-up [2,3]. The recently developed Contegra^® ^valved bovine conduit (Medtronic Inc., Minneapolis, MN, USA) has encouraged short-term success in experimental animal studies [4,5], as well as in humans [6,7]. It consists of a bovine jugular vein, which contains a venous valve with three leaflets that open to allow the forward flow of blood and close to prevent the backward flow of blood, and it functions like the patient's natural pulmonary artery valve.

The Contegra^® ^Pulmonary Valved Conduit can be used in children and young adults under the age of 18 [8]. Isolated endocarditis of the pulmonary valve is uncommon and usually occurs in conjunction with tricuspid and/or left-sided valvular endocarditis [9]. A mortality rate for Staphylococcus aureus prosthetic valve endocarditis is very high [10].

We describe here a case of a 20 years old male patient with acute endocarditis of bovine Contegra valved conduit, four years after right ventricular outflow tract reconstruction and atrial septal defect correction, associated with acute glomerulonephritis, renal failure and severe anemia, secondary to methicillin-resistant Staphylococcus aureus (MRSA) infection.

## Case presentation

A 20 years old male patient admitted to our clinic complaining of fever, sweating, fatigue and lost of weight. He was referred by the secondary health centre in Gjakova, after ten days hospitalization for suspicious endocarditis. Four years ago he underwent atrial septal defect correction and implantation of pulmonary valve with Contegra^® ^Pulmonary Valved Conduit in Lausanne, Switzerland. A murmur on the precordium was prescribed on his third month of life. During all his life he was enabled for enforced physical activity. He periodically complained of fatigue, dispnea and later on the cyanosis appeared. These signs and symptoms were aggravated on his age of 15^th^. At that time (four years ago) he was diagnosed and successfully operated for dysplastic pulmonary valve with its severe stenosis and severe regurgitation, severe dilatation of the right ventricle, pulmonary trunk and right pulmonary artery, as well as the type II atrial sepal defect.

On admission the blood pressure was 130/80 mmHg, pulse rate 100 beats/min., and body temperature 39°C. Heart auscultation revealed regular heart rhythm, clear sounds and systolic murmur 5/6 on the precordium with punctum maximum on the second right intercostal space. On lung auscultation there was normal findings. ECG showed normal sinus rhythm, heart rate of 100 beats/min., and right axis deviation.

Laboratory data showed: high erythrocite sedimentation (132 mm/h), low number of erythrocites (2.94 × 10^12^/mm^3^); low hemoglobin rate (8 g/dL), low hematocrite (24%), high number of leucocytes (19.7 × 10^3^/mm^3^, high percentage of granylocites (82.7%), high urea (27 mmol/L) and creatinine (860 mmol/L) concentrations. All other laboratory findings were within normal reference range. Blood cultures revealed the MRSA infection.

Chest X-ray showed mild pleural adhesions in right diaphragmal localization, and the triangled heart shadow. The abdominal ultrasonographic examination showed splenomegaly and signs of diffuse glomerulonephritis.

**Figure 1:**
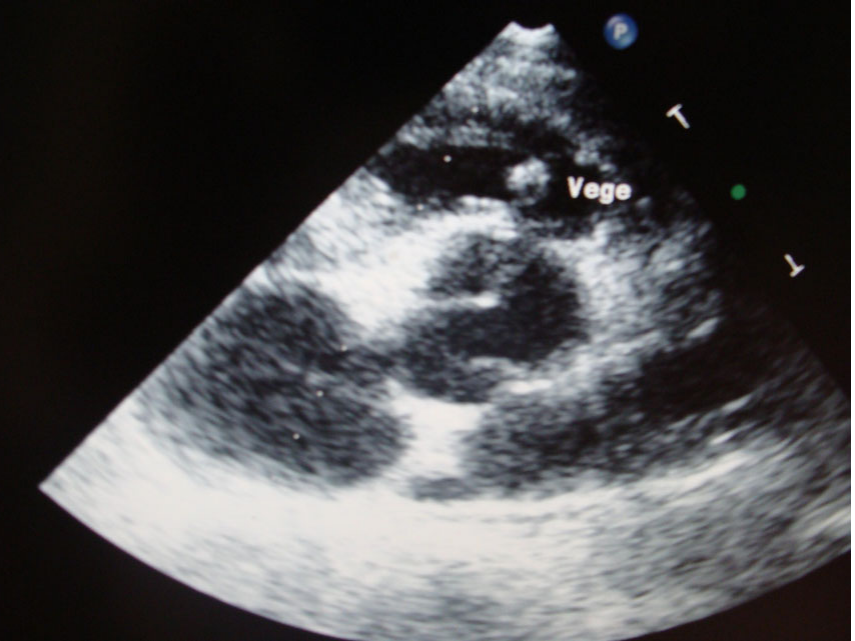
**The presence of the huge vegetation on the Contegra bovine leaflet, with dimensions 0.56 × 0.75 cm, in short-axis parasternal view of transthoracic echocardiography**.

**Figure 2:**
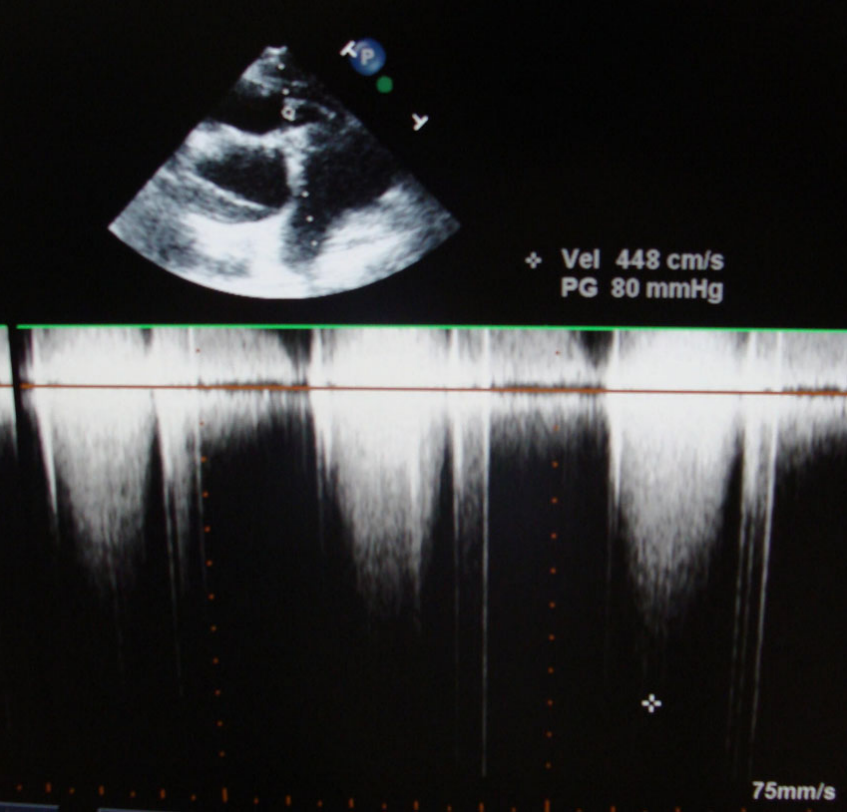
**Increased pressure gradient (maximal pressure gradient of 80 mmHg) of the Contegra valve assessed by continuous wave Doppler in short-axis parasternal view of transthoracic echocardiography**.

**Figure 3:**
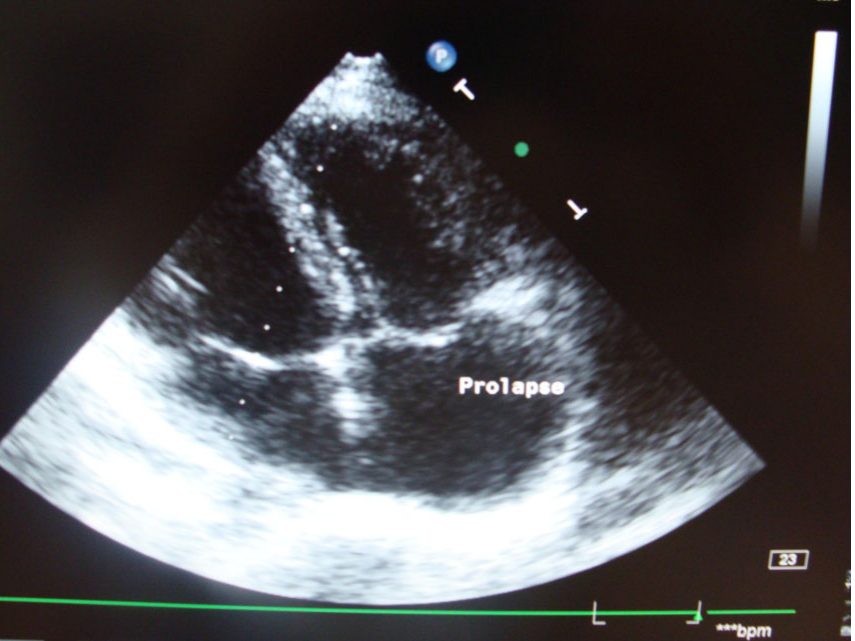
**Thickend tricuspid valved, with its important prolapse, in apical four-chamber view of transthoracic echocardiography**.

**Figure 4:**
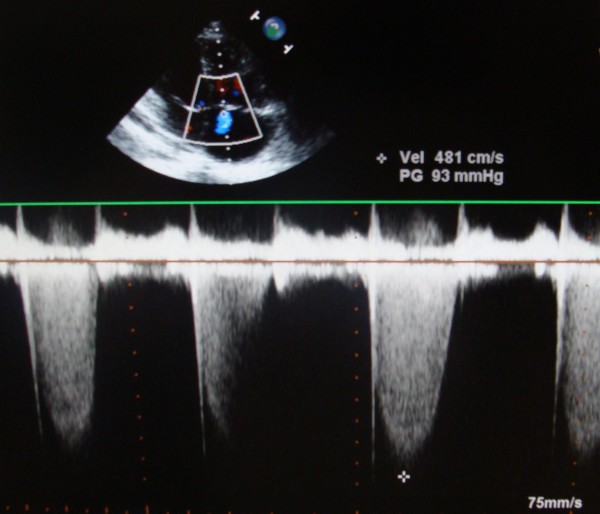
**Severe tricuspid regurgitation and important pressure gradient (93 mmHg), assessed by continuous wave Doppler in apical four-chamber view of transthoracic echocardiography**.

Transthoracic echocardiography demonstrated the presence of the huge vegetation on the Contegra bovine leaflet, with dimensions 0.56 × 0.75 cm (Fig. 1). Continuous wave Doppler assessment of the Contegra valve showed increased pressure gradient (maximal pressure gradient of 80 mmHg, Fig. 2). Tricuspid valve was thickened, with its important prolapse (Fig. 3). Severe tricuspid regurgitation, high trans-tricuspid pressure gradient (93 mmHg, Fig. 4) and enlarged right heart chambers (right ventricle = 5.2 cm, right atrium = 6.9 cm) were registered.

The left atrium (3.6 cm), aortic root (3.1 cm), left ventricular dimensions (end systolic = 5.4 cm, end-systolic = 3.7 cm), thickens (interventricular septum = 0.9 cm, left ventricular posterior wall = 0.8 cm) and left ventricular systolic function (shortening function = 31%, ejection fraction = 59%) were within normal range. It was registered mild mitral regurgitation through mildly thickened mitral valve leaflets.

The patient was treated with beta-blockers, salycilates, antibiotics (Vancomycin and Gentamycin, which was reduced and stopped, because of renal failure that was aggravated during the hospitalization). Also, the transfusions with red blood cells were administrated for the anemia correction.

After three weeks of the intensive treatment, the patient was transferred to the cardiovascular center that performed the operation four years ago, for the re-correction of the pulmonary artery valve, in general stable conditions.

## Discussion

Since introduction in 1999, Contegra^® ^valved bovine conduit (Medtronic Inc., Minneapolis, MN, USA), which consists of a bovine jugular vein, is successfully used in patients for the right ventricular outflow tract reconstruction in, and it has advantages compared with homo- or porcine xenografts [6,7].

Right-sided infective endocarditis represents about 5–10% [11], whereas the pulmonary valve endocarditis is less than 2% [12] of all cases of endocarditis, and usually it is accompanied with other congenital heart anomalies.

The results of Contegra bovine conduit in problematic right ventricular and pulmonary artery are encouraging, with good long term survival [6,13-15]. Endocarditis of the Contegra conduit was shown very rare in the follow-up of these patients. Breyman T, et al. [6], followed-up the highest number of patients that underwent this correction (71 patients) and did not register any case with endocarditis, as well as other researchers [13-15].

Shebani S, et al. [16] followed 64 patients operated with Contegra conduit, and they detected one patient that had endocarditis early after operation and did not survive. By our knowledge this is the only case reported as the endocarditis of Conegra conduit in pulmonary valve.

We present case of a 20 years old male patient with acute endocarditis of bovine Contegra valved conduit, four years after right ventricular outflow tract reconstruction and atrial septal defect correction, associated with acute glomerulonephritis, renal failure and severe anemia, secondary to MRSA infection. We consider that our case is a complex one, not referred before in the literature.

A major limitation of our case presentation is the lack of trans-esophageal echocardiography, which we decided to don't perform, according to the severe general conditions of the patient in the admission and their improvement during the hospitalization, that justified our diagnosis based on trans-thoracic echocardiography.

In conclusion, we present a complex patient with acute endocarditis of bovine Contegra valved conduit, associated with acute glomerulonephritis, renal failure and severe anemia, secondary to MRSA infection.

## Consent

Written informed consents were obtained from the patients for publication of this case report and accompanying images. Copies of the written consents are available for review by the Editor-in-Chief of this journal.

## Competing interests

The authors declare that they have no competing interests.

## Authors' contributions

GB, RO, ID, FA, ZV and MB analyzed and interpreted the patients' data. GB, ID and FA performed transthoracic echocardiography. GB was a major contributor in writing the manuscript. All authors read and approved the final manuscript
